# Inhalation of honey reduces airway inflammation and histopathological changes in a rabbit model of ovalbumin-induced chronic asthma

**DOI:** 10.1186/1472-6882-14-176

**Published:** 2014-05-29

**Authors:** Nurfatin Asyikhin Kamaruzaman, Siti Amrah Sulaiman, Gurjeet Kaur, Badrul Yahaya

**Affiliations:** 1 Regenerative Medicine Cluster, Advanced Medical and Dental Institute, Universiti Sains Malaysia, Kepala Batas, Penang 13200, Malaysia; 2 Department of Pharmacology, School of Medical Sciences, Universiti Sains Malaysia, Kubang Kerian, Kelantan 16150, Malaysia; 3 Institute for Research in Molecular Medicine, Universiti Sains Malaysia, Minden, Penang 11800, Malaysia

## Abstract

**Background:**

Honey is widely used in folk medicine to treat cough, fever, and inflammation. In this study, the effect of aerosolised honey on airway tissues in a rabbit model of ovalbumin (OVA)-induced asthma was investigated. The ability of honey to act either as a rescuing agent in alleviating asthma-related symptoms or as a preventive agent to preclude the occurrence of asthma was also assessed.

**Methods:**

Forty New Zealand white rabbits were sensitized twice with mixture of OVA and aluminium hydroxide on days 1 and 14. Honey treatments were given from day 23 to day 25 at two different doses (25% (v/v) and 50% (v/v) of honey diluted in sterile phosphate buffer saline. In the aerosolised honey as a rescue agent group, animals were euthanized on day 28; for the preventive group, animals were further exposed to aerosolised OVA for 3 days starting from day 28 and euthanized on day 31. The effects of honey on inflammatory cell response, airway inflammation, and goblet cell hyperplasia were assessed for each animal.

**Results:**

Histopathological analyses revealed that aerosolised honey resulted in structural changes of the epithelium, mucosa, and submucosal regions of the airway that caused by the induction with OVA. Treatment with aerosolised honey has reduced the number of airway inflammatory cells present in bronchoalveolar lavage fluid and inhibited the goblet cell hyperplasia.

**Conclusion:**

In this study, aerosolised honey was used to effectively treat and manage asthma in rabbits, and it could prove to be a promising treatment for asthma in humans. Future studies with a larger sample size and studies at the gene expression level are needed to better understand the mechanisms by which aerosolised honey reduces asthma symptoms.

## Background

Asthma is a chronic inflammatory airway disease that affects around 300 million people worldwide. According to a survey conducted in 2006, the prevalence of asthma among Malaysian adults was about 4.5%, and the rate was increasing, especially among children [1]. Asthma accounted for 0.4% of all deaths worldwide, and the number is increasing annually. In fact, this disease has become a significant cause of morbidity and mortality in developed countries [2].

Asthmatic patients usually experience recurrent episodes of wheezing, breathlessness, chest tightness, and coughing, especially at night and early in the morning [3]. The disease is characterised by variable degrees of chronic inflammation and airway remodelling. Airway remodelling refers to alteration of the structure of the airway, which includes epithelium, mucosa, and submucosa, leading to thickening of the airway wall [4]. Other important features are goblet cell hyperplasia and metaplasia, increased smooth muscle mass, bronchial gland enlargement, angiogenesis, and alteration of the extracellular matrix components [5,6]. Both goblet cell metaplasia and bronchial gland enlargement lead to airway mucus hypersecretion, which contributes to airway obstruction [6].

Asthma is thought to occur due to over-expression of Th2 cytokines such as interleukin (IL)-4, IL-5, and IL-13 [3,7]. Over-expression of IL-4 leads to eosinophilia and mucus metaplasia as well as subepithelial fibrosis [7], whereas IL-5 over-expression is thought to result in an increased number of eosinophils in the airway, which is the hallmark of asthma [3]. IL-13 over-expression causes subepithelial fibrosis, mucus metaplasia, and induced infiltration of eosinophils and macrophages into the airways [6,8]. Over-expression of these Th2 interleukin molecules results in airway hyperresponsiveness (AHR) [7].

Drug therapy has become the most common treatment to manage asthma. Examples include short acting β-agonists, inhaled corticosteroids, and long acting β-agonists. These drugs alleviate asthmatic attacks by relaxing the airway smooth muscle. However, asthma attacks and exacerbations can still occur, as the treatment does not alter the underlying pathology [9]. Unfortunately, prolonged use of drug therapy can cause local and systemic side effects, including oral candidiasis, dysphonia, growth failure, accelerated loss of bone mass, elevated intraocular pressure, and mild tachyphylaxis [10]. These problems highlight the need to find an alternative treatment for asthma with fewer side effects, and the use of natural products is a promising approach.

Honey has been widely used as a remedy for treating ailments such as cough, fever, infections, and inflammation. It is reportedly has antioxidant, anti-immunomodulatory, and antibacterial effects [11,12]. Although honey is usually taken orally, inhalation is a more suitable way to administer honey to treat asthma; it would ensure that the maximum amount of honey is deposited in the airways to treat the disease. Maksoud and Rahman [13] showed that honey nebulisation was effective in treating upper acute asthma in paediatric patients. However, there have been no comprehensive studies examining the effect of nebulised honey (i.e., aerosolised) on pathophysiological changes of the airway tissues in association with asthma-related features such as on goblet cell hyperplasia, mucus over-production, and inflammatory cell responses.

The traditional oral administration of honey routes it towards the digestive system, therefore delaying its efficacy in treating patients with an acute asthma attack. Thus, the goal of the present study was to investigate the effect of aerosolised honey on histopathological changes of the airway in a rabbit model of ovalbumin (OVA)-induced chronic lung disease, which mimics the human condition of asthma. Experiments were conducted to determine whether aerosolised honey can act as a rescue agent by reducing asthma-related symptoms and/or as a preventive agent by preventing the occurrence of asthma.

## Methods

### Ethics

This study was conducted in strict accordance with university guidelines. The protocol was approved by the Animal Ethics Committee of Universiti Sains Malaysia (USM/Animal Ethics Approval/2012/(77)(379)).

### Animals

**Figure 1:**
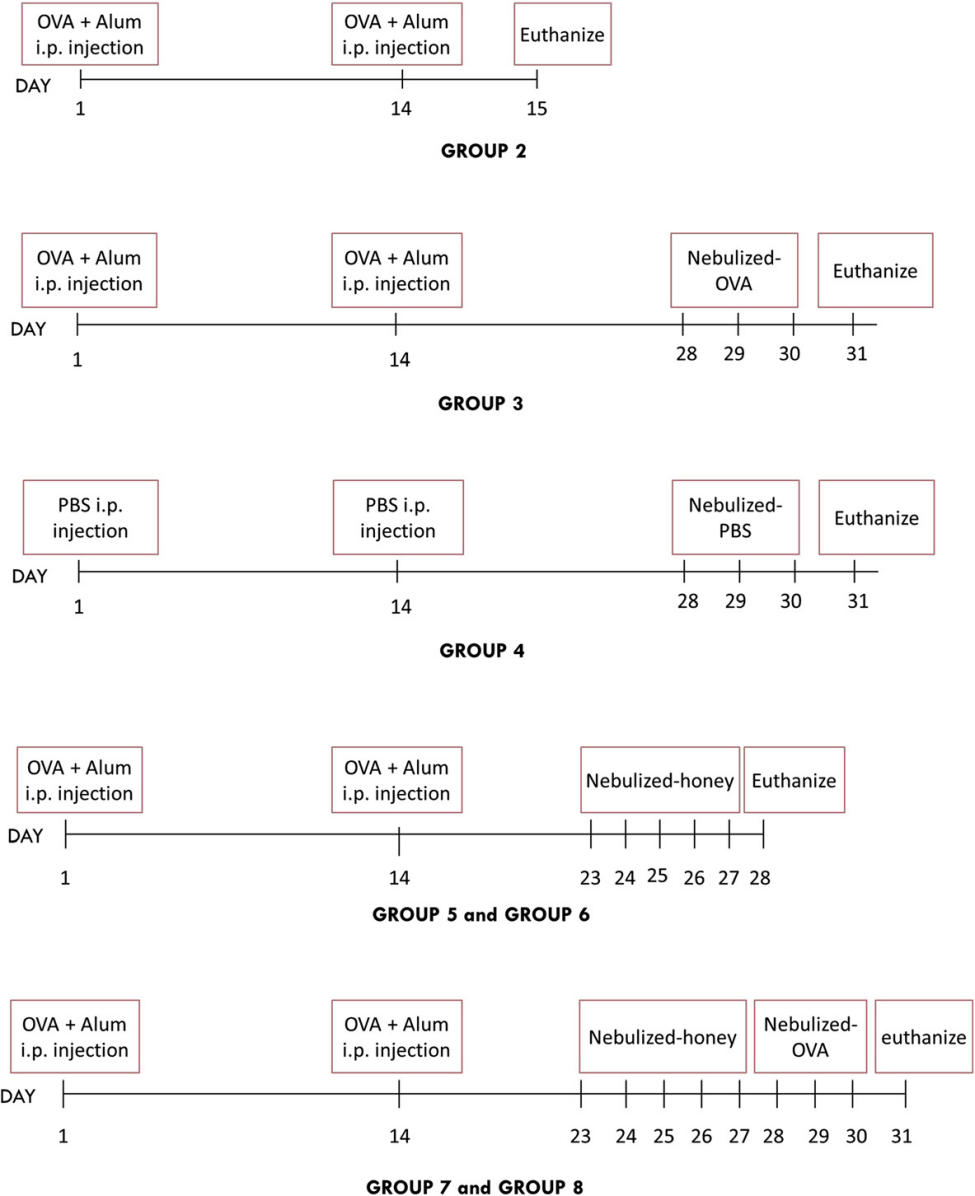
**Procedural timeline.** Schematic diagrams showing the procedures and timelines for OVA induction of airway inflammation in the rabbit model and for treatment with aerosolised honey.

Forty rabbits (breed: New Zealand white, *Oryctolagus cuniculus*; sex: male = 37, female = 3; weight: 2.40 ± 0.56 [mean, standard deviation, SD]) were used in this study. The rabbits were housed in an air-conditioned room, kept on a 12 hour light/dark cycle, and had access to clean food and water.Animals were divided into eight groups of five rabbits each. Control groups were treated as follows: group 1, naïve (normal); group 2, injected intraperitoneally (i.p.) twice with OVA at days 1 and 14; group 3, i.p. injection twice with OVA at the same time points, followed by aerosolised OVA for three consecutive days at day 28 to day 30; group 4, treated twice with i.p. phosphate buffered saline (PBS) at days 1 and 14 followed by aerosolised PBS for three consecutive days at day 28 to day 30 (the negative control). Asthma was induced in rabbits in the other groups twice via i.p. injection of OVA at days 1 and 14, and they then were treated as follows: groups 5 and 6, honey at 25% (v/v) and 50% (v/v), respectively, treatment with aerosolised-honey for five consecutive days from day 23 to day 27, followed by euthanization, to test for rescue effects; groups 7 and 8, treatment with aerosolised honey at the same concentrations and time period, followed by aerosolised OVA for three consecutive days from day 28 to day 30, before euthanized on the following day to test for preventative effects – Figure 1.

### Experimental protocol

Experiments were followed protocols adopted from El. Gazzar et al. [14]. Induction of allergic airway inflammation was performed by i.p. sensitization and airway challenge through nasal inhalation (Figure 1). Animals in group 2 were sensitized (i.p.) on days 1 and 14 with 0.1 mg of OVA (Sigma A5503; Sigma-Aldrich, St. Louis, MO, USA) and 10 mg of aluminium hydroxide (alum) (donated by the EMAN Testing and Research Laboratory, School of Pharmaceutical Sciences, Universiti Sains Malaysia) in 2 ml of PBS. Twenty-four hours after the second sensitization, animals in group 2 were sacrificed. Animals in the aerosolised OVA group (group 3) were further challenged with aerosolised OVA (10 mg/ml) for 3 consecutive days starting from day 28 until day 30. Aerosolised OVA was generated using an ultrasonic nebuliser (MabisMist^TM^, Mabis Healthcare Inc, USA). Rabbits in the negative control group (group 4) were treated with both i.p PBS and aerosolised PBS. Rabbits in groups 3 and 4 were euthanized at day 31 using intravenous injection of sodium pentobarbital (1 ml/kg body weight) (Dolethal, Lure Cedex, France).

For the honey treatment groups, asthma was first induced via i.p. OVA in rabbits in groups 5, 6, 7, and 8. They then were treated for 20 min with 5 ml of aerosolised *Tualang* honey (Federal Agricultural Marketing Authority (FAMA) of Malaysia). Honey was diluted with PBS to either 25% (v/v) or 50% (v/v). Animals in groups 5, 6, 7 and 8 were treated with 25% (v/v) and 50% (v/v) honey. Treatment was given daily starting from day 23 until day 27. Rabbits in groups 5 and 6 (models of honey as rescue agent) were euthanized on day 28, whereas rabbits in groups 7 and 8 (honey as preventive agent) were subsequently challenged with aerosolised OVA (10 mg/ml) starting from day 28 until day 30 and euthanized at day 31. Figure 1 outlines of the study procedures and time points for each group.

### Collection of bronchoalveolar lavage (BAL) fluid

Twenty four hours after the final treatment, rabbits were anaesthetised and lungs were lavaged three times by instillation and withdrawal of 3 ml of sterile PBS through an endotracheal tube. BAL fluid from each animal was placed in sterile plastic tubes, cooled in ice, and centrifuged (1200 × *g*) at room temperature for 5 min. The cell pellets were resuspended in 200 μl of sterile PBS. Total cell counts were determined using a Neubauer hemocytometer (Marienfeld, Germany). A 10 μl aliquot of the cell suspension was mixed with 10 μl of Trypan blue (Gibco-Life Technologies, NY, USA) solution to allow counting of nucleated cells. For differential cell counts, 100 μl of the cell suspension were applied to a slide by cytospinning at 500 rpm for 5 min followed by staining with Wright-Giemsa solution (Sigma-Aldrich).

### Histological and morphometric analyses

The right lung of each rabbit was fixed in 10% (v/v) formalin overnight and processed according to standard histological protocols. Tissues ultimately were embedded in paraffin (Paraplast® Plus, Leica Biosystems, Ayer Rajah, Singapore) and cut into 3 μm sections with a rotary microtome (Accu-Cut®, SRM™ 200, Sakura®, CA, USA). The sections were stained with haematoxylin and eosin (H&E) (Sigma-Aldrich) to examine the histology of airways and cellular infiltration into peribronchial connective tissues [15] and with Alcian Blue (AB; Merck, Darmstadt, Germany) and Periodic Acid Schiff (PAS; Sigma-Aldrich) to detect mucus-secreting goblet cells.

The slides stained with H&E (n = 5 for each group) were analysed for airway tissue structure and morphometric features such as thickness of epithelium, mucosal, and submucosal regions. For these measurements, photomicrographs were taken using a DP72 Olympus digital camera (Tokyo, Japan) attached to an Olympus BX53 microscope. Measurements were taken from five different points on larger airways for each slide.

To measure the effect of honey on mucus production, the numbers of mucus-secreting goblet cells were counted. The goblet cells were examined in five different fields in three large lung sections from each animal (n = 5 for each group). Results were calculated as the number of positively stained goblet cells within 100 epithelial cells. All slides were blinded prior to morphometric analysis.

### Statistical analyses

Our preliminary results showed that there was no statistically significant difference in terms of the degree of inflammatory responses and airway epithelial structures in both naïve (group 1) and PBS-treated (group 4) groups (data not shown); therefore, these two groups were combined and served as the control animal group. The i.p. OVA (group 2) and i.p. + aerosolised OVA (group 3) groups were combined and were later known as the OVA-induced injury group.

For statistical analyses, results were compared among groups to determine the following: (1) the effect of aerosolised honey on airway structures and the number of mucus-secreting cells; (2) the effect of aerosolised honey as rescue and preventive agents; (3) the effect of treatment with 25% (v/v) and 50% (v/v) honey; and (4) the effect of treatment with 25% (v/v) and 50% (v/v) honey for both rescue and preventive groups.

Comparisons between groups were made using independent t-tests, and the data are presented as means (SD). A *p* value of < 0.05 was considered to be significant, whereas *p* < 0.001 was considered to be highly significant. Statistical analysis was performed using SPSS Statistics software version 20 (IBM, NY, USA).

## Results

### Aerosolised honey reduced the inflammatory cell response

**Figure 2:**
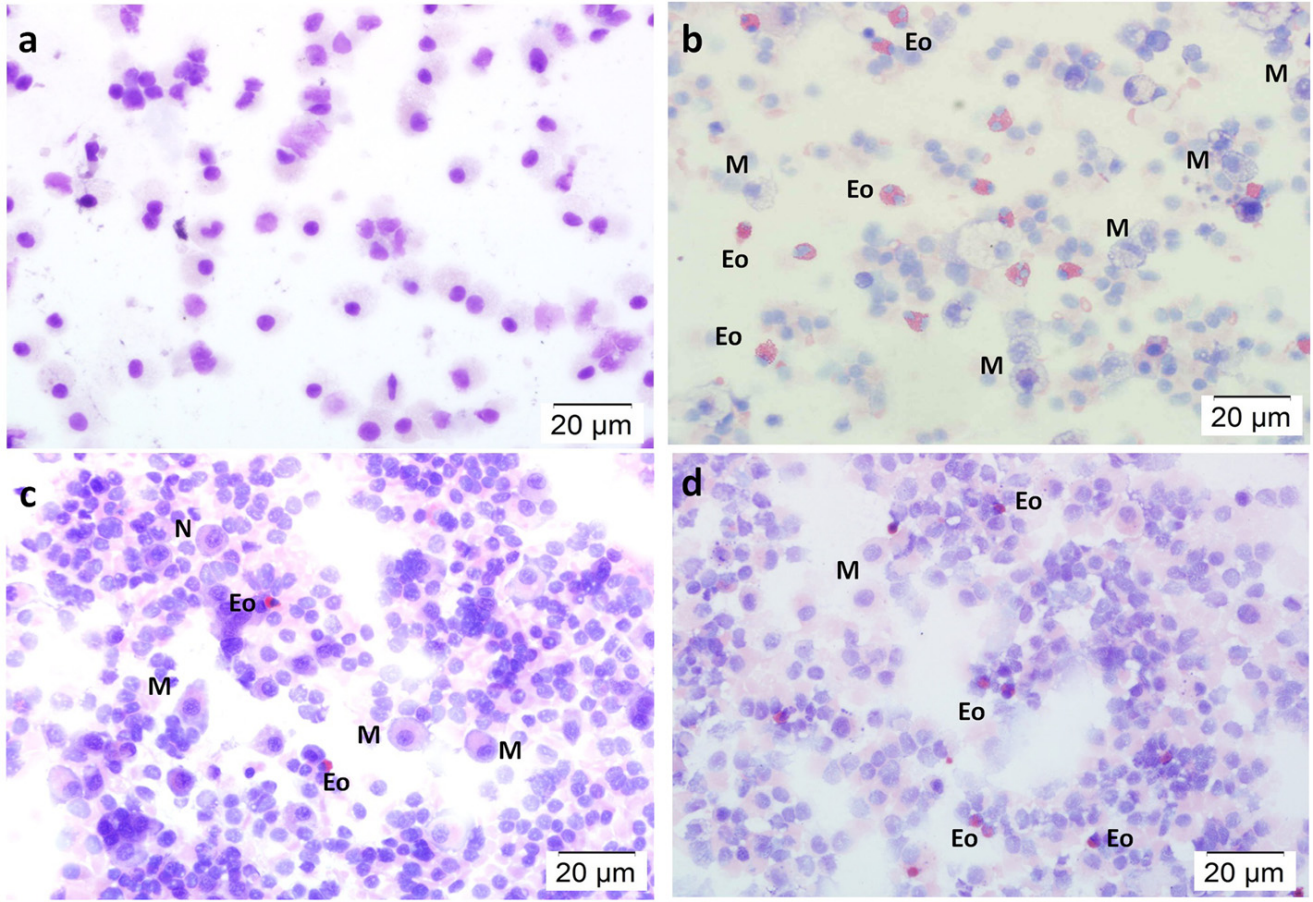
**Effect of aerosolised honey on inflammatory cell response.** The panels show Wright-Giemsa stained BAL fluid from the control group **(a)**, injury group **(b)**, and 50% honey-treated group (rescue, **c**; preventive, **d**). Rabbits in the aerosolised OVA group (group 3 – OVA-induced injury) resulted in greater infiltration of neutrophils (N), eosinophils (Eo), and macrophages (M), whereas the number of infiltrated inflammatory cells was low (control animal group). Treatment with aerosolised honey reduced the infiltration of these inflammatory cells. However, further exposure to OVA following treatment elicited the second phase of the inflammatory reaction.

Figure 2 shows the representative microscopic images of cytospun BAL fluid stained with Wright-Giemsa, and Table 1 summarises the histopathological examination of the effect of aerosolised honey on inflammatory cell response. Exposure to OVA led to the infiltration of inflammatory cells into the airway, such as eosinophils, mononuclear cells (lymphocytes and plasma cells), neutrophils, and macrophages (Figure 2b). OVA-induced asthmatic rabbits were characterised by dense peribronchial inflammatory cell infiltration, especially eosinophils (which are the hallmark of asthma). The accumulation of eosinophils in the BAL fluid was reduced by treatment with aerosolised honey (Figure 2c). However, further exposure to aerosolised OVA in the honey preventive groups led to an increase in infiltration of inflammatory cells (Figure 2d). Based on the finding as shown in the Table 1, the honey treatment in the rescue group shows less numbers of inflammatory cells present as compared to honey treatment in the preventive group, with 50% of honey gave a better effect. Nevertheless, this result shows that treatment with aerosolised honey can reduce the inflammatory cell response.

**Table 1:** Summary of the effect of aerosolised honey on inflammatory cell response

**Group**	**Inflammatiory cells**			
	**Eosinophils**	**Mononuclear**	**Neutrophils**	**Macrophage**
Control	-	-	-	-
OVA-induced injury	+++	+++	++	+++
25% Honey rescue	++	+++	+	++
25% Honey preventive	+	+	-	+
50% Honey rescue	-	-	-	+
50% Honey preventive	+	+	-	++

Indicator: -(normal), +(mild), ++(moderate), +++(dense).

### Aerosolised honey had a significant effect on airway structures

**Figure 3:**
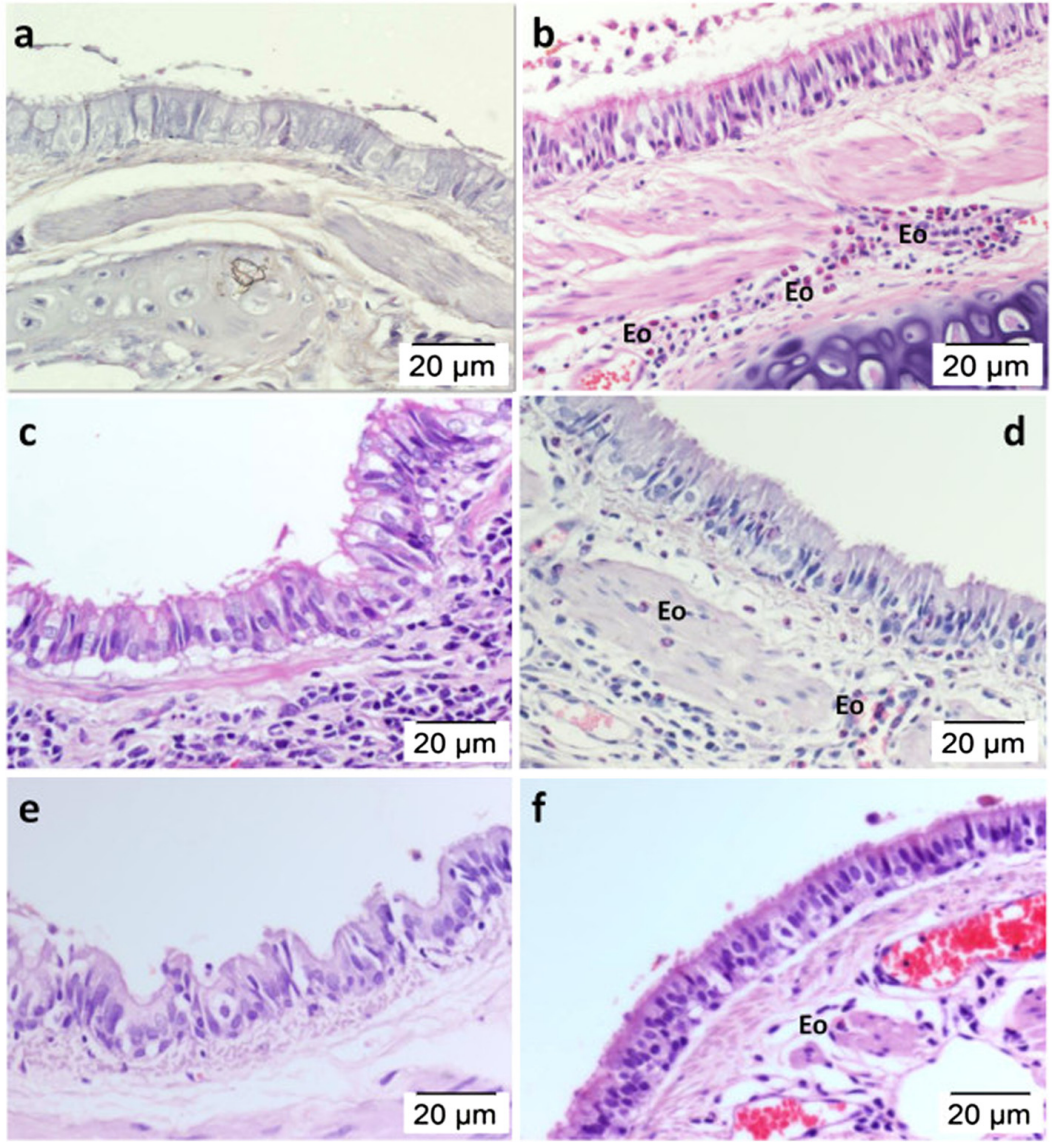
**Effect of aerosolised honey on airway structures in the OVA-induced asthma rabbit model.** The panels show H&E stained lung tissues from the control group **(a)**, OVA-induced injury group **(b)**, 25% honey-treated group (rescue, **c**; preventive, **d**), and 50% honey-treated group (rescue, **e**; preventive, **f**). Exposure of the lungs to OVA led to epithelial hypertrophy and peribronchial inflammatory cell infiltration **(b)**. Treatment with aerosolised honey for three consecutive days following OVA sensitization and before the OVA challenge (honey rescue groups) markedly attenuated airway inflammation **(c and e)**. However, the subsequent OVA challenge (honey preventive groups) caused epithelial hypertrophy as well as peribronchial cell infiltration to occur **(d andf)**.

OVA treatment led to the infiltration of inflammatory cells into the peribronchial region of the lungs (Table 1). The histological appearance of the airway structures in the injury group differed compared to the morphology of the airway in the control group. Figure 3 shows representative microscopic images of tissue sections from different groups stained with H&E. The structure of the epithelial layer was normal and peribronchial cell infiltration was absent in lung sections from the control animal group (Figure 3a). OVA sensitization and repeated OVA challenge caused thickening of the airway wall, including the epithelium, mucosa, submucosa, and smooth muscle. It was also associated with epithelial folding and epithelial cell shedding, elongation of cellular epithelial nuclei, and goblet cell hyperplasia, which causes accumulation of mucus in the lumen of the bronchioles (Figure 3b). Compared to the OVA-induced injury group, aerosolised honey at both the 25% and 50% doses in the rescue groups reduced the infiltration of inflammatory cells in the peribronchial region (Figure 3c, e). In the preventive groups with both doses of honey, epithelial thickening was increased compared to that of the control group (Figures 3d, f).

**Figure 4:**
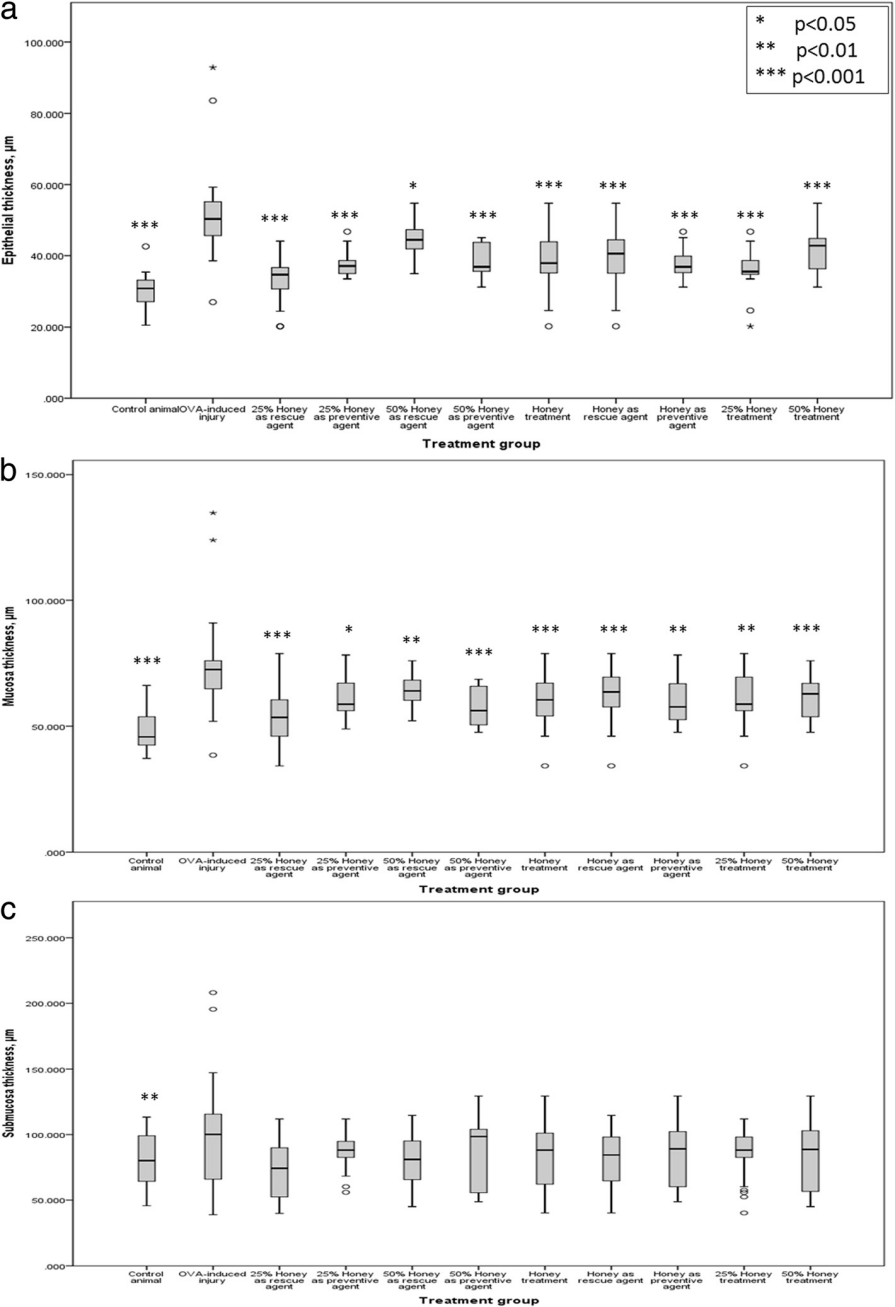
**Effect of aerosolised honey treatment on airway structure.** The three parameters measured were **(a)** epithelial thickness, **(b)** mucosa thickness, and **(c)** submucosa thickness. Statistical analyses were performed to compare: 1) the control animal group with the OVA-induced injury group and 2) the OVA-induced injury group with different treatment groups. *p* < 0.05 was considered to be statistically significant.

Figure 4 shows (1) OVA induced the development of epithelial hyperplasia and thickening of mucosal and submucosal regions and (2) the effect of treatment with 25% and 50% aerosolised honey on airway structures following OVA treatment. Treatment with aerosolised honey at both concentrations in both the rescue and preventive experiments restored the airway structures following induction with OVA (Table 2). In all four treatment groups, honey treatment was significantly reduced the thickness of the airway epithelial and mucosal regions (p < 0.05) following asthma induction with OVA. However, inhalation of honey did not significantly restore the submucosal region (p > 0.05).

**Table 2:** Statistical summary of the effect of aerosolised honey on airway structure

**Treatment Group**	**Lung Structure**	**p-value**
Control Animal vs OVA-induced Injury***	Epithelial Thickness	0.000
Control Animal vs OVA-induced Injury***	Mucosal Thickness	0.000
Control Animal vs OVA-induced Injury**	Sub Mucosal Thickness	0.003
OVA-induced Injury vs 25% Honey as Rescue Agent***	Epithelial Thickness	0.000
OVA-induced Injury vs 25% Honey as Rescue Agent*	Mucosal Thickness	0.018
OVA-induced Injury vs 25% Honey as Rescue Agent#	Sub Mucosal Thickness	0.219
OVA-induced Injury vs 50% Honey as Rescue Agent*	Epithelial Thickness	0.036
OVA-induced Injury vs 50% Honey as Rescue Agent**	Mucosal Thickness	0.009
OVA-induced Injury vs 50% Honey as Rescue Agent#	Sub Mucosal Thickness	0.155
OVA-induced Injury vs 25% Honey as Preventive Agent***	Epithelial Thickness	0.000
OVA-induced Injury vs 25% Honey as Preventive Agent**	Mucosal Thickness	0.006
OVA-induced Injury vs 25% Honey as Preventive Agent#	Sub Mucosal Thickness	0.326
OVA-induced Injury vs 50% Honey as Preventive Agent***	Epithelial Thickness	0.000
OVA-induced Injury vs 50% Honey as Preventive Agent***	Mucosal Thickness	0.000
OVA-induced Injury vs 50% Honey as Preventive Agent#	Sub Mucosal Thickness	0.211
OVA-induced Injury vs Honey as Rescue Agent***	Epithelial Thickness	0.000
OVA-induced Injury vs Honey as Rescue Agent**	Mucosal Thickness	0.002
OVA-induced Injury vs Honey as Rescue Agent#	Sub Mucosal Thickness	0.104
OVA-induced Injury vs Honey as Preventive Agent***	Epithelial Thickness	0.000
OVA-induced Injury vs Honey as Preventive Agent***	Mucosal Thickness	0.000
OVA-induced Injury vs Honey as Preventive Agent#	Sub Mucosal Thickness	0.168
OVA-induced Injury vs Honey Treatment***	Epithelial Thickness	0.000
OVA-induced Injury vs Honey Treatment***	Mucosal Thickness	0.000
OVA-induced Injury vs Honey Treatment#	Sub Mucosal Thickness	0.081

*p < 0.05.

**p < 0.01.

***p < 0.001.

#p > 0.05.

### Effect of aerosolised honey on goblet cells

**Figure 5:**
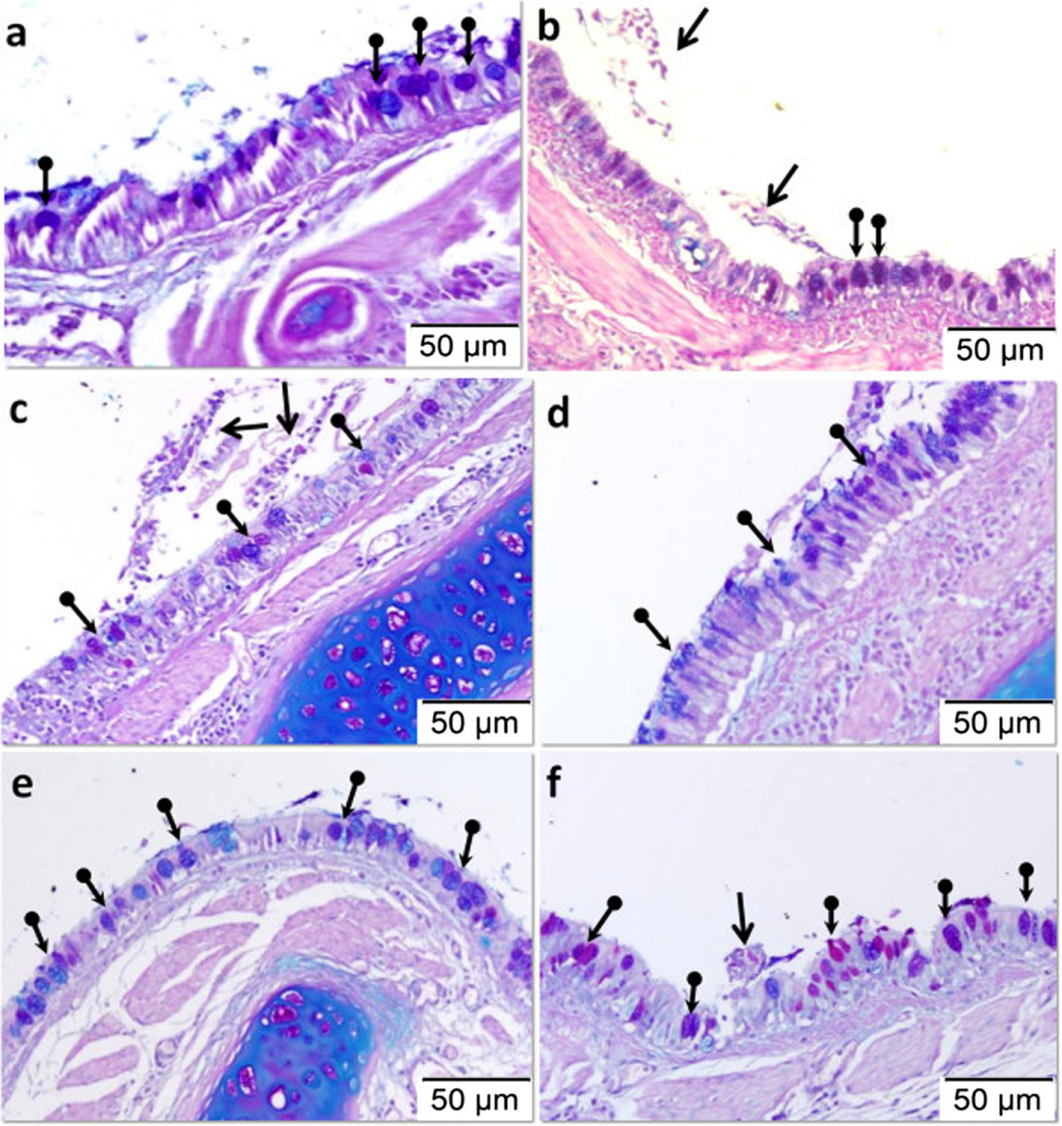
**Effect of aerosolised honey treatment on goblet cell hyperplasia.** The panels show AB-PAS stained lung tissues from the control group **(a)**, injury group **(b)**, 25% honey-treated group (rescue, **c**; preventive, **d**), and 50% honey-treated group (rescue, **e**; preventive, **f**). Arrows in the figure indicate the accumulation of mucus in the airway lumen, and round-ended arrows indicate representative images of goblet cells.

**Figure 6:**
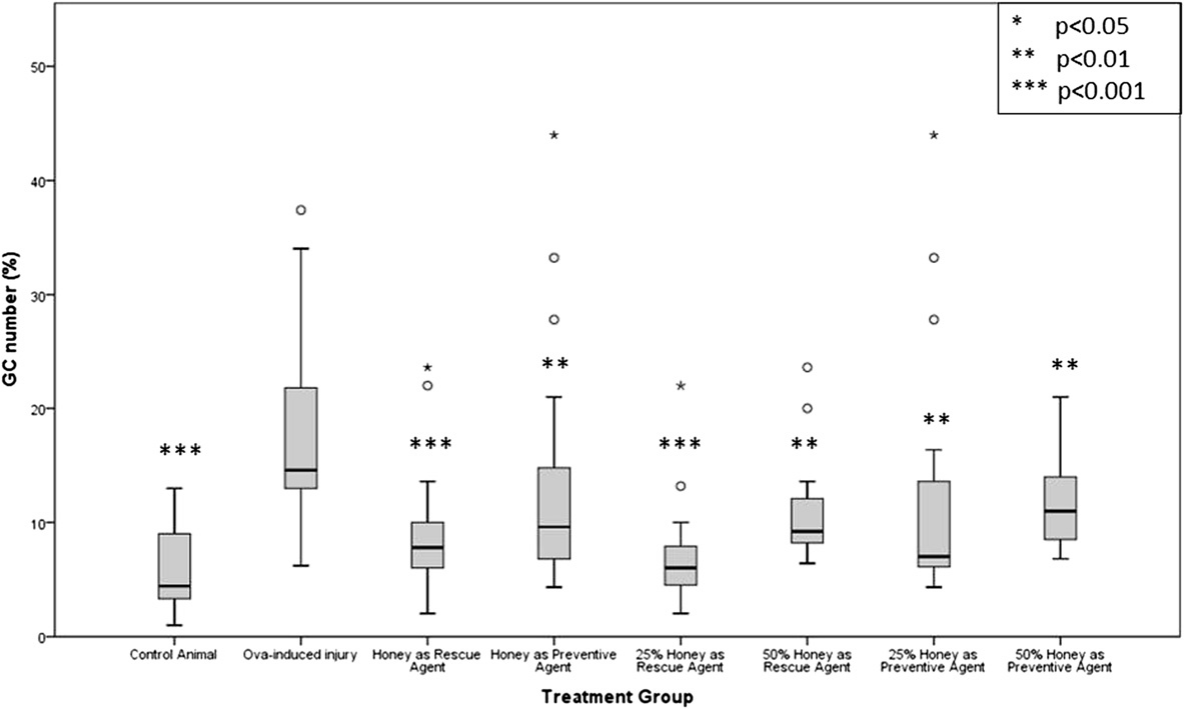
**Effect of aerosolised honey treatment on goblet cell numbers.** Results of morphometric analysis of the effect of OVA induction on goblet cell numbers as compared to control animals and the effect of aerosolisation of 25% and 50% honey in different treatment groups on goblet cell numbers following OVA-induced injury. *p* < 0.05 is considered to be statistically significant.

Figure 5 shows representative microscopic images of lung sections stained with AB-PAS. Figure 6 shows the results of morphometric analysis of the effect of OVA induction on goblet cell hyperplasia as compared to the control and aerosolisation of 25% and 50% honey on goblet cell numbers in different treatment groups. Following OVA sensitization and challenge, AB-PAS positive goblet cells were significantly more abundant in the OVA-induced injury group compared to the control group (Figure 6; Table 3) (p < 0.001). Following honey inhalation, the numbers of goblet cells were significantly reduced in all treatment groups (p < 0.05) (Figure 6; Table 3).

**Table 3:** Statistical summary of the effect of aerosolised honey on goblet cell numbers

**Treatment Group**	***p*-value**
OVA-induced Injury vs Control Animal	0.000
OVA-induced Injury vs 25% Honey as Rescue Agent	0.000
OVA-induced Injury vs 25% Honey as Preventive Agent	0.008
OVA-induced Injury vs 50% Honey as Rescue Agent	0.001
OVA-induced Injury vs 50% Honey as Preventive Agent	0.006

## Discussion

The aim of the present study was to investigate the effect of aerosolised honey as an agent to alleviate the asthma-related histopathological changes that occur in the rabbit airway following OVA-induced injury. The effects of aerosolised honey on inflammatory cell response, histopathological changes of lung tissues, and goblet cell hyperplasia were assessed. Although many studies have used mice as a model, we used the rabbit as our model system to imitate human chronic lung disease because it is phylogenetically closer to humans and offers a better understanding of lung structure than smaller animal models [16].

After asthma was induced by OVA, honey was administered in the form of aerosol particles through a nebuliser. This technique was selected because it ensures maximum deposition of honey on the surfaces of the airway. The aerosolised honey particles generated by a nebuliser are between 1 to 5 μm in size [17]. Adjei et al. [18] reported that particles must be less than 3 μm in order to be deposited in the pulmonary zone of the lungs. Thus, aerosol particles generated by a nebuliser are small enough to reach the lungs and provide the optimal effect on the subjects. In comparison to smaller animals, findings from other study showed that delivery of aerosol via nasal inhalation led to deposition of more than 80% of the aerosol particles in the nasal region [19], thus hindering the effectiveness of the treatment.

The pathogenesis of asthma begins with alteration in the structure of the airway components, such as thickening of the epithelial and subepithelial regions [5], epithelial folding and desquamation [4,6], goblet cell metaplasia, enlargement of blood vessels [5], increased infiltration of inflammatory cells, and excessive mucus secretion into the airways [20]. The results of our study showed that the OVA sensitization and airway challenge led to thickening of the epithelial layer, mucosa, and submucosa of the lung tissues, indicating that OVA treatment effectively established the model for airway remodelling in rabbits.

In this study, aerosolised honey was used to investigate the capability of honey to 1) alleviate the effects of asthma (i.e., a rescue treatment) and 2) assess the potential for honey to prevent the occurrence of asthma (i.e., a preventive agent). In general, aerosolised honey treatment (both 25% and 50% for both rescue and preventative treatments) resulted in a significant decrease in the thickening of the epithelial and mucosal regions. However, the submucosal region was not affected by the honey treatment. Thickening of the mucosal regions depends on thickening of the epithelial layer, whereas the submucosal region of the rabbit lung contains no (or less) smooth muscle and submucosal glands – are the main contributors for submucosal thickening. In summary, both the 25% and 50% honey treatments significantly alleviated airway inflammation following induction of injury with OVA, and this was true for both the rescue and preventive experiments. This finding suggests that honey may prove useful in alleviating asthma-associated histopathological changes as well as preventing the development of asthma upon subsequent induction with an allergen.

In the present study, some of the lung tissue samples for all groups showed signs of epithelial desquamation. This feature is often considered to be a pathological feature of asthma based on observation of post-mortem specimens and endobronchial biopsies from asthmatic patients [7,21]. However, epithelial shedding has also been reported from bronchial biopsy specimens from healthy nonasthmatic subjects [22]. Hence, the presence of epithelial desquamation may be related to the sampling technique used [7], and it may have occurred during collection of the BAL fluid samples in this study. Infiltration of inflammatory cells into the peribronchial region is thought to be a pivotal process in the pathogenesis of asthma. In response to injury, Th2 cells are activated and produce a large amount of IL-4, IL-5, and IL-13. These cytokines are responsible for recruiting inflammatory cells such as eosinophils and macrophages into the bronchial region. Thus, eosinophilic inflammation is the pathological hallmark of asthma, as the cells act as effectors in allergic airway disease by releasing cytotoxic granule proteins [15,23].

OVA-induced asthma results in chronic airway inflammation and characteristically is associated with infiltration of lymphocytes, eosinophils, macrophages, and neutrophils into the peribronchial region [24]. In our study, treatment with aerosolised honey decreased the number of inflammatory cells, especially eosinophils, in the airway region (Figure 2c; Table 1). Treatment with 25% honey in the rescue group was more effective at suppressing the infiltration of eosinophils than treatment with 25% honey in the preventive group. This suggests that treatment with honey at 25% concentration effectively reduced the inflammatory cell response. In addition, inhalation of honey prevented the occurrence of asthma in the model system. The number of infiltrating cells in the 50% dosage group was lower than that of the 25% dosage group in the asthma prevention experiment.

The anti-inflammatory effect of honey in both *in vivo* and *in vitro* systems has been described previously. However, most of these studies focused on the role of honey in wound healing. Bashkaran et al. [25] assessed the anti-inflammatory and antioxidant properties of *Tualang* honey in treating alkaline injury to the eyes of rabbits and reported that honey contained an anti-inflammatory component with an effectiveness comparable to that of conventional treatment. *In vitro* studies showed that honey types such as *Gelam*[25], *Manuka*[26], and *buckwheat*[27] have anti-inflammatory components. *Manuka* honey was reported to increase the expression of the pro-inflammatory cytokine TNF-α as well as anti-inflammatory cytokines such as IL-10, IL-1ra, and growth factors PDG and TGF-β [26]. Kassim et al. [28] suggested that the anti-inflammatory properties of honey may be attributable to its phenolic compounds.

Goblet cells are responsible for secreting mucus on the surface of the airway. Airway mucus protects the epithelial surfaces from injury and facilitates the removal of bacterial, cellular, and particulate debris from the lung [29]. In the case of asthma, goblet cells become hyperplastic, and a high proportion of goblet cells and submucosal gland enlargement lead to mucus hypersecretion and contribute significantly to airflow limitation [6,29]. As shown in Table 3 and Figure 6, treatment with aerosolised honey (regardless of dosage and its mechanism of action) significantly inhibited (p < 0.05) goblet cell hyperplasia and over-production of mucus following OVA induction. Thus, the results indicate that aerosolised honey may have an inhibitory effect on the development of asthma. The ability of aerosolised honey to eliminate goblet cell hyperplasia and mucus over-production suggest that honey treatment has great potential in the management of patients with asthma, both in terms of prevention of disease and rescuing patients from symptoms.

## Conclusion

Herein we demonstrated that treatment with honey at both concentrations tested effectively inhibited OVA-induced airway inflammation by alleviating asthma-related histopathological changes in the airway and also prevented the occurrence of asthma. Inhalation of honey was also found to effectively eliminate mucus-secreting goblet cell hyperplasia. Future studies of these effects at the gene expression level are needed to better understand the mechanisms by which aerosolised honey reduces asthma symptoms.

### Financial disclosure

This study was supported by the E-Science Fund Grant Scheme from the Ministry of Science, Technology and Innovation (MOSTI), Malaysia (305/CIPPT/613224).

## Competing interests

The authors report no conflicts of interest. The authors alone are responsible for the content and writing of the paper.

## Authors’ contributions

Conceived and designed the experiment: NAK, SAS, BY. Performed the experiment: NAK. Analyzed the data: NAK, GK, BY. Wrote the first draft of the manuscript: NAK, BY. Contributed to the writing of the manuscript: NAK, GK, BY. Read and approved with manuscript results and conclusions: NAK, SAS, GK, BY.

## Pre-publication history

The pre-publication history for this paper can be accessed here:

http://www.biomedcentral.com/1472-6882/14/176/prepub
